# Trace Elements in Marine Sediment and Organisms in the Gulf of Thailand

**DOI:** 10.3390/ijerph15040810

**Published:** 2018-04-20

**Authors:** Suwalee Worakhunpiset

**Affiliations:** Department of Social and Environmental Medicine, Faculty of Tropical Medicine, Mahidol University, 420/6 Ratchavithi Rd, Bangkok 10400, Thailand; suwalee.wor@mahidol.ac.th; Tel.: +66-2-354-9100

**Keywords:** trace element, environment, pollution, sediment, gulf of Thailand

## Abstract

This review summarizes the findings from studies of trace element levels in marine sediment and organisms in the Gulf of Thailand. Spatial and temporal variations in trace element concentrations were observed. Although trace element contamination levels were low, the increased urbanization and agricultural and industrial activities may adversely affect ecosystems and human health. The periodic monitoring of marine environments is recommended in order to minimize human health risks from the consumption of contaminated marine organisms.

## 1. Introduction

Environmental pollution is an urgent concern worldwide [1]. Pollutant contamination can exert adverse effects on ecosystems and human health [2]. Trace elements are one type of pollutant released into the environment, and metal contamination levels are rising. The main sources of trace elements are natural activities such as volcanic eruptions and soil erosion, and human activities such as industrial production, waste disposal, the discharge of contaminated wastewater, the inappropriate management of electronic waste (e-waste), and the application of fertilizers in agriculture [3,4,5,6,7].

Once trace elements are released into the environment, they can be dispersed by the wind and deposited in soil and bodies of water, accumulating in marine sediments [8,9]. Moreover, trace elements can enter the food chain and accumulate in the human body, sometimes at harmful levels [10]. The most common trace elements found in the environment are cadmium (Cd), chromium (Cr), copper (Cu), nickel (Ni), arsenic (As), lead (Pb), zinc (Zn), and mercury (Hg) [10,11,12].

**Figure 1:**
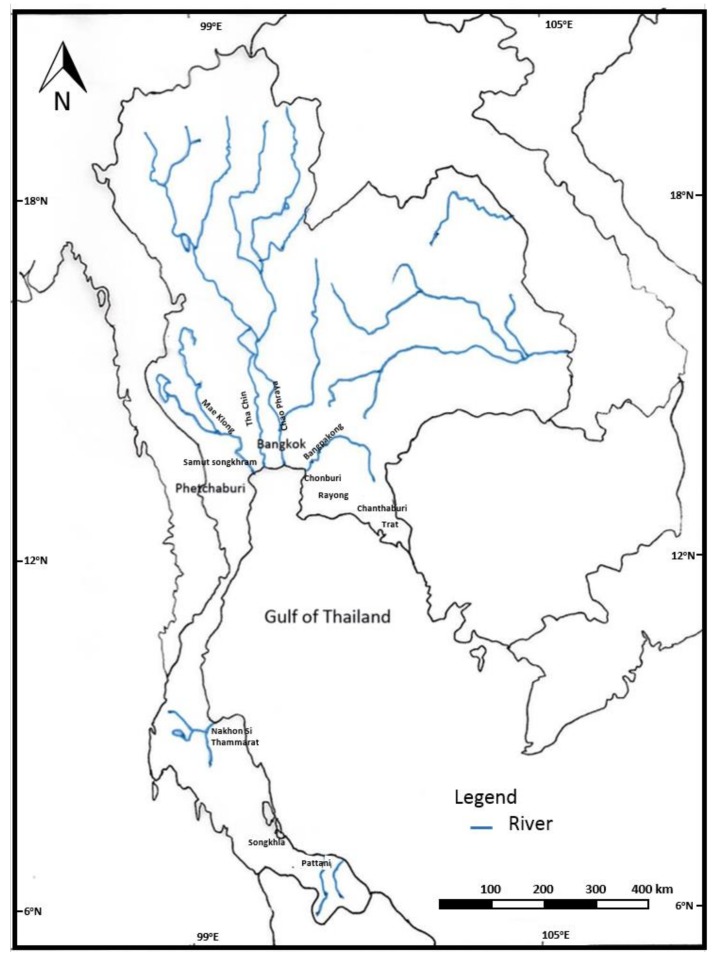
Map of the Gulf of Thailand.

The Gulf of Thailand is an enclosed body of water in the southwestern part of the South China Sea that is situated between latitudes 5°00′ and 13°30′ N and longitudes 99°00′ and 106°00′ E [13,14]. The Gulf is bound by the line from Cape Cà Mau in Vietnam to the coastal city of Kota Bharu on the east coast of Peninsular Malaysia [15]. The average depth is 45 m, with a maximum depth of 80 m at the center of the Gulf [16]. The Gulf can be divided into two parts: Upper and Lower. The Upper Gulf, at the innermost area, is an inverted U-shape that is characterized as an estuary of a drowned river valley that was once above the surface. Fresh water from four major rivers—Mae Klong, Tha Chin, Chao Phraya, and Bangpakong—and minor rivers from the eastern and western parts of Thailand flows into the Upper Gulf, supplementing the nutrients, sediments, and pollutants in the basin [17]. The map of the Gulf of Thailand is shown in Figure 1.

Trace element contamination in the Gulf of Thailand, which is caused by rapid economic, social, agricultural, and industrial development and urbanization over the last three decades, has emerged as a major concern. Trace element concentrations and distribution in marine environments, especially enclosed marine environments such as the Upper Gulf, can be affected by factors such as variations in agricultural practices, population growth, industrial activities, land cover, land use, urbanization, seasonal changes, and climate change [5]. Specifically, industrial development projects such as the Eastern Seaboard have been suspected to contribute to trace element contamination in the Upper Gulf [18], which is known as a major marine resource in terms of fishing and aquaculture; its pollution will adversely affect its environmental qualities and increase health risks from consuming contaminated marine organisms, especially commercially important aquatic food sources such as fish, crabs, and mollusks [17]. Although the Thai government has established laws and regulations aimed at pollution control, the environmental quality of the Upper Gulf of Thailand is still a matter of debate, requiring scientific investigation.

The objective of this review was to evaluate published research on trace element concentrations in marine sediment and organisms, in order to reflect the status of trace element pollution in the Gulf of Thailand.

## 2. Trace Elements in Marine Sediment in the Gulf of Thailand

When the Eastern Seaboard Development Project was initiated in 1980, core sediment was sampled from the Upper Gulf of Thailand for trace elements. Concentrations of aluminum (Al), Cd, Cr, Pb, and Zn were determined from 5 cm strata of sediment cores, and high concentrations of Cd and Pb were found in surface sediment collected from the Chao Phraya estuary [19]. In 1984, sediment cores were sampled again to assess sedimentation rates using a ^210^Pb method, and to determine thesource of element input in the Gulf. Research found that only a small amount of sediment was transported by the major rivers to the Upper Gulf and distributed in the Lower Gulf; the sediment accumulation rate ranged between 4–11 mm/year. In addition, the concentrations of Cd, cobalt (Co), and Pb in the Upper Gulf of Thailand at that time were distinctly controlled by natural inputs [20].

Shazili et al. measured the trace metal concentrations in sediment in the Gulf of Thailand before and after the monsoon period that occurred between September 1995 and April 1996 [21]. Mean concentrations of Cu and Pb increased significantly, while concentrations of Cd, Cr, iron (Fe), and Zn decreased. The study also found that trace element concentrations in the sediment were higher in sampling sites nearer the shore. This finding may be attributable to the closer anthropogenic inputs of the areas and higher silt and clay contents of sediments near land that result in a higher binding affinity of the elements. Meanwhile, Ratasuk et al. [22] investigated Cu adsorption to marine sediment in areas near the shore, and found a high capacity (in the range of 1.5–4.0 µg/kg Cu). The higher the organic matter and clay content, the higher the binding capacity of Cu; salinity was not found to exert an effect on Cu adsorption by sediments. Furthermore, the findings indicated that the Cu that partitioned into sediment in this coastal region was not likely to be easily released into the water phase, and that the sediment in the Samut Prakan coastal region would rapidly adsorb the Cu with little return into the water column. Similarly, Potipat et al. [23] found the levels of Cd, Cr, Cu, Fe, Pb, and Zn were influenced by the organic matter content and grain sizes of clay particles in the sediments. In addition, Liu et al. [24] assessed trace element contamination in 157 surface sediments collected from the western Gulf of Thailand, and concluded that sediment composition was the main factor controlling the levels of trace elements, while hydrodynamic conditions, and especially seasonal variations, influenced the distribution of metals. These studies demonstrated the influence of organic matter content on the variation of trace elements distribution as observed in other coastal regions around the world [25].

The Pollution Control Department in Thailand conducted a monitoring program between 1998–2001 to determine the levels of trace elements in marine sediment that was collected from the coast. The highest concentrations of As, Cd, and Hg in marine sediment were found at the Chao Phraya estuary (12.0 µg/kg, 0.2 µg/kg, and 1.8 µg/kg, respectively). As and Cd levels did not exceed the standard limits for marine sediment, but Hg concentrations did [26]. After those trace element concentrations in sediments were collected from the northwestern Gulf of Thailand, Censi et al. surveyed the coastal area affected by the Mae Klong River and Phetchaburi River [27]. The result showed that Cr, Cu, Ni, and Zn concentrations in the sediment exceeded the standards of NOAA [28] as well as those of Australia and New Zealand [29] in some sampling areas. Besides, the authors concluded that Cr, Cu, and Zn occurred preferentially in the colloidal fraction, while Ni occurred mainly in the dissolved fraction. This revealed the influence of sediment characteristics and sediment-water-sediment partitioning on the distribution patterns of trace elements and the level of contamination that was observed before in other studies [30]. Rermdumri et al. [31] sampled sediment from 13 locations in the Upper Gulf of Thailand in April, August, and December of 2007, and found that mean concentrations of Cd, Cu, Pb, and Zn, were 2.05 mg/kg, 16.21 mg/kg, 28.19 mg/kg, and 64.07 mg/kg, respectively, and only Cd levels exceeded the standard values set by NOAA [28] and by Australia and New Zealand [29]. These levels were lower than those reported by previous studies [26]. After that, Thongra-ar et al. [5] investigated the trace elements in sediments along the Eastern Coast of the Gulf of Thailand, which is an area with heavy industrial development. The study measured levels of Cd, Cu, Fe, Hg, manganese (Mn), Ni, Pb, and Zn; it found that most metals did not exceed the standard values of NOAA [28] and Australia and New Zealand [29], with the exception of Pb, Cu, and Ni. Pb contamination was high at some locations, including the Map Ta Phut Industrial Estate in Rayong Province, where multiple petrochemical factories are located.

In regards to the expansion of the industrial sector and urbanization, a huge volume of wastewater has been discharged into the river, which ultimately flows to the estuary. Ei Tun et al. [32] studied the influence of wastewater from industrial and domestic sources on Cd concentrations in the bottom sediment of the Upper Gulf of Thailand. Sediment with finer particle size fractions (<0.075 mm) collected from the Chao Phraya River mouth, Bangpakong River mouth, and Klong Dan estuary contained higher levels of Cd than sediment with coarser fractions at all sampling stations. In addition, Khidkhan et al. [33] investigated trace element contamination in sediments from the Don Hoi Lot sandbar, which is a tidal flat located at the mouth of the Mae Klong River in Muang District, Samut Songkhram Province. The river receives wastewater from various sources, leading to the accumulation of pollutants in the sandbar. The study found that Fe was the predominant contaminant, and that metal levels fluctuated between the wet and dry seasons. A subsequent sampling found a non-significant increase in Cd, Fe, Mn, Ni, and Pb [34].

Sediments in mangrove areas have also been studied. Buajan and Pumijumnong [35] measured the levels of Cd, Cu, Pb, and Zn in mangrove sediments at the Tha Chin Estuary of Samut Sakhon Province, and found that levels were higher in the dry season than in the wet season. In addition, metal concentrations were higher at sampling sites closer to the mainland, and gradually decreased with proximity to the Gulf. The physical and chemical characteristics of the sediment may influence trace element concentrations. Sediment with higher percentages of organic matter is expected to contain higher levels of trace elements, especially Cu and Zn, because organic matter affects binding capacity. Sediment with finer particles is expected to contain higher levels of Cd and Zn, as its greater surface area has a high adsorption capacity. Thongra-ar et al. [36] investigated the levels of Cd, Cu, Fe, Hg, Pb, Ni, and Zn in the mangrove sediments of Ban Laemchabang, Chon Buri Province, which is located near an urban community and three petroleum refineries. Metal concentrations decreased as the distance from the urban and industrial zones of activity increased. In addition, the concentrations of trace elements in the sediments correlated positively with organic matter, silt, and clay contents. This finding is in agreement with those of various studies on the effect of organic matter, silt, and clay on the binding capacity of trace elements; generally, metals are more likely to bind to fine particles [37].

Since trace element contamination in marine sediment is caused by various human activities, the relationship between land use and metal accumulation has also been studied. Sowana et al. [38] reported that Pb levels were higher in residential, industrial, and dockyard areas, and lower in shrimp farms, agricultural land, and mangrove forests. The major sources of Pb in the study area were both natural and anthropogenic. Abandoned tin mines were the major source of Pb around the Pattani River, although the contamination level was low; boat repairs and industrial activities were the dominant sources of Pb at the Pattani River mouth. Meanwhile, Potipat et al. [23] collected sediment samples from areas representing industrial, urban, and agricultural activities; they found that conservative land use in areas affected by urbanization yielded sediment samples with the highest mean concentrations of metals. Besides, Sudsandee et al. [39] monitored the concentrations of trace elements in sediment collected from the Bangtaboon Estuary, Tha Chin Estuary, and Mueang Chon Buri coastal area in the Upper Gulf, and found that the mean concentrations of Cd, Cr, Cu, Hg, Mn, Ni, Pb, and Zn were lower than NOAA recommendations [28]. This study also concluded that spatial and temporal variations in levels of trace elements in marine sediments may be attributable to land-based activities.

The 2011 Thailand floods have been studied as a putative factor in trace element deposition in sediments. Mingkhwan et al. collected sediment samples from the Upper Gulf in July 2012 [40], and found that As, Cd, Cu, Hg, Pb, and Zn concentrations in sediments were relatively low, and the floods did not affect marine sediment quality. However, sediments in areas adjacent to the major river mouths were Pb-enriched, and the degree of contamination decreased in a seaward direction, implying that the Pb originated in anthropogenic activities.

In another study, marine sediments were collected from 25 areas in 10 provinces along the inner and eastern Gulf of Thailand between February and April of 2016. These tested for seven trace elements [41], and As, Cr, Cu, Pb, and Zn concentrations exceeded NOAA standards [27], especially in the Eastern Gulf. There were spatial variations in the levels of all of the metals tested; these differences may have been caused by both natural and human activities. However, a rapid expansion of the industrial and urban sectors in the Rayong and Chonburi Provinces appears to be the major cause.

Recently, Khowhit and Chunkao [42] determined the concentration of trace elements in Laem Phak Bia, Phetchaburi Province, the receiving area of treated wastewater from the lagoon of The King’s Royally Initiated Laem Phak Bia Environmental Research and Development Project. Mean concentrations of As, Cd, Cr, Hg, Ni, and Pb did not exceed the standard values recommended by NOAA marine guidelines [27]. Although the sediment at the receiving area of the wetland effluent contained more silt than the receiving area of the lagoon effluent, trace element levels in the sediment did not differ significantly. This may be attributable to the effect of metal input from human activities such as mining [43]. Some results of trace elements in marine sediment are summarized in Table 1.

## 3. Trace Elements in Marine Organisms in the Gulf of Thailand

Trace element contamination in marine environments can also exert adverse effects on organisms and the humans that consume them; the Gulf of Thailand is a significant source of seafood, so levels of trace elements in marine organisms are a relevant concern. Several species of marine organisms have been investigated for concentrations of the following trace elements.

The levels of Cd, Cr, Cu, Pb, and Zn in green mussels (*P. viridis*) collected from the Upper Gulf were assessed by Hungsapreugs and Yuangthong [44]; they found that concentrations of Cr and Cu in mussels were higher in the flood season than in the rainy season. This probably due to the influence of lower salinity on metal accumulation [45]. Meanwhile, Sukasem and Tabucanon [46] collected *P. viridis* from 10 locations along the coast, and reported that levels of Cd in mussels from the Western Coast of the Gulf were higher than in mussels from other parts. However, none of the mussels contained metals in levels exceeding the acceptable limits set by the Ministry of Public Health, Thailand [47].

*P. viridis* mussels were also collected and tested for metals by Ruangwises and Ruangwises [48], who found that levels of Cd, Cr, Ni, and Pb were higher in mussels collected from the Upper Gulf than in mussels collected from the Lower Gulf, while Cu, Mn, and Zn levels did not differ significantly by sampling site. The authors noted that the concentrations of some trace elements in the mussels collected from the Upper Gulf were higher than those reported by an earlier study [44], although the levels were still within the acceptable range for human consumption.

The concentrations of Cd, Cu, Pb, and Zn in the edible marine organisms blood cockle (*A. granosa*), blue swimmer crab (*Portunus pelagicus*), mullet (*Liza dussumieri*), and northern whiting (*Sillago sihama*) collected from a wastewater treatment area in Phetchaburi Province over several seasons were investigated [49]. Zn concentrations were substantially higher in all of the samples, and metal levels were highest in blue swimmer crab. No significant differences were found between the samples and those collected from a reference site, with the exception of Zn levels in *A. granosa* that was collected during the rainy season and the southwest monsoon. Overall, all of the levels were within the acceptable ranges for human consumption. *A. granosa* samples were also collected from the mouth of the Bangpakong River, in the coastal area of Chachoengsao Province, Samut Prakan Province, and Chonburi Province [50]; mean levels of Cd, Pb, Hg, and Zn did not exceed the food compliance limits set by the Ministry of Public Health, Thailand [47], and did not differ between farmed and natural-habitat cockles, although seasonal variations were observed.

*P. viridis* and *A. granosa* from the Ban Laem district of Phetchaburi Province, an important aquaculture area for both wild-caught and farmed seafood, were assessed regarding their levels of Cd, Cr, Ni, and Pb [51]. Seasonal variations in Ni and Pb levels were observed, with a notable increase during the rainy season. Pb levels in both organisms were slightly higher than the permissible limits of trace elements in food set by the Ministry of Public Health, Thailand [47]. Furthermore, Khowhit et al. studied the effect of wastewater effluent on trace element accumulation in marine organisms [52] through collecting edible mollusks from the coastal area of the Laem Phak Bia Sub District and Ban Laem District of Phetchaburi Province, which is an area that receives treated wastewater from the Phetchaburi municipal wastewater treatment system. Concentrations of As, Cd, Cr, Hg, Ni, and Pb were analyzed in the tissues of *A. granosa*, *Katelysia hiantina*, *K. marmorata*, *Marcia hiantina*, *M. marmorata*, and *Phaxas attenuatus*. The highest concentrations were of As in *K. marmorata*, while the lowest concentrations were of Hg in *M. hiantina*. *K. marmorata* samples contained the highest concentrations of five of the six metals analyzed in the study. Nonetheless, no levels exceeded the recommended standards [47]. After that, Khowhit et al. examined trace elements in the hard clam *M. meretrix* [53]. The levels of As, Cd, Cr, Hg, Ni, and Pb in all of the samples were within the permissible limits for consumption. In a subsequent study, Khowhit and Chunkao [42] reported the levels of trace elements in *Meretrix* spp. collected from mudflat areas in Phetchaburi Province where treated domestic wastewater was discharged. No significant differences in metal levels were observed between hard clams from the wastewater area and clams from a lagoon treatment system, and all of the metals that were detected were below the permissible limit.

Cd, Hg, Pb, and tin (Sn) levels in *P. viridis* and *A. granosa* collected from the inner Gulf of Thailand between May and August 2009 were determined [54]. The results found that metal levels did not exceed the standards mandated by the Ministry of Public Health, Thailand [47] and the European Union [55], with the exception of Cd in farmed *A. granosa*. This agree with the study of Pasanum et al. [56] that collected *A. granosa* and *P. viridis* from coastal farms and pond farms in three provinces bordering the Upper Gulf to determine their Cd, Hg, Pb, and Sn contents. In some *P. viridis* samples, Cd was higher than the levels recommended by the European Union [55], and one sample of *A. granosa* exceeded Thailand Ministry of Public Health standards for Pb [47]. The study suggested that *A. granosa* and *P. viridis* from the inner Gulf of Thailand contained acceptable levels of trace elements, but that pond farm sediment may accumulate Pb and Cd [54]. In addition, Thongra-ar et al. [57] reported the concentrations of Cd, Cu, Hg, Pb, and Zn in seafood samples from the coastal area of Map Ta Phut Industrial Estate in Rayong Province. Eighty-four percent of samples did not exceed permissible limits of trace elements in food [47], and the samples that did exceed the limits for Cd, Cu, and Zn came from shellfish.

Another study looked at Cd, Cu and Zn concentrations in edible fish, squid, shrimp, and crab from fishing areas along the coast of Songkhla Province; levels of Cd, Cu, and Zn were highest in *Octopus* spp., while the highest concentration of Pb was found in *Metapenaeus* spp. [58].

Potipat et al. [23] investigated the accumulation of Cd, Cr, Cu, Fe, Pb, and Zn in the tissues of the oyster *Saccostrea cucullata* and of *P. viridis* collected from a coastal area receiving runoff and sewage from three rivers: the Wang-Ta-Nord, the Chanthaburi, and the Welu of Chanthaburi Province. Most of the metal levels were lower than permissible limits, with the exception of Cu and Zn concentrations in the oysters.

Six species of edible mollusks in the coastal area of Leam Klat, Mueang District, Trat Province (*Meretrix casta*, *M. meretrix*, *Solen grandis*, *Solen regularis*, *Solen strictus*, and *Pseudodon* sp.) were examined for As, Cd, Cr, Hg, Ni, and Pb concentrations. As concentrations were highest, but no levels exceeded the metal safety standards for human consumption [59].

Pradit et al. [60] reported metal concentrations in the cockles *A. granosa* and *A. inaequivalvis* collected from Pattani Bay in February and September of 2014. The highest concentrations of Cd and Cu were found in February, while the highest concentration of Zn was found in September. Cd and Pb concentrations of cockles collected in September exceeded permissible limits [47,55], leading to concerns regarding a possible health risk to consumers. Boat repairs were indicated as a potential source of Pb contamination, together with natural weathering and human activities along the Pattani River. Sudsandee et al. [61] studied concentrations of multiple metals in blood cockles from the Bangtaboon Estuary, Tha Chin Estuary, and the Mueang Chon Buri coast, but did not find excessive concentrations. However, metal levels varied significantly between sampling sites, depending on local land-based activities. Some results of trace elements in marine organisms are summarized in Table 2.

## 4. Trace Elements: Sources and Potential Human Health Risk

Trace elements have been defined as elements that occur in natural and pertubated environments in small amounts [62,63,64,65]. Although trace elements occur naturally, environmental contamination by trace elements has been accelerated through human activities such as mining, smelting, refinery, industrial production, and pesticides used in agriculture [10]. Some trace elements such as Cu, Mn, Ni, and Zn are essential elements for physiological and biological functions in the human body, but exposure to excess amounts can cause adverse effects. The possible human health hazards of major trace elements regarding the reviewed are as the following.

Cd is found in the Earth’s crust, and is commonly bound with Zn, Pb, or Cu ores. Human activities can generate cadmium to atmosphere, such as fossil fuels and municipal waste burning [63]. Cd can accumulate in soil, water, sediment, and aquatic organisms. Chronic oral exposure can cause adverse effects on the kidney, liver, lung, bone, immune system, blood, and nervous system [64].

Cr is widely distributed in rocks or soil, and has been used in various human activities such as trivalent chromium (Cr^+3^) or hexavalent chromium (Cr^+6^); these forms are used for chrome plating, dyes and pigments, leather, wood preservation, and the cooling treatment of tower water. Once Cr is released to the atmosphere, it will deposit on soil and water by wet or dry deposition, and can accumulate in aquatic organisms [65]. Human exposure to Cr^+6^ through food and water consumption can contribute adverse effects to the liver, kidney, gastrointestinal, immune systems, and possibly the blood [65,66] In addition, Cr^+6^ has been grouped as a human carcinogen [66].

Cu is found in the Earth’s crust and has been used in the production of cables, electric equipment, printed circuits, industrial metal planting and coating, and copper wires. It can enter the environment through waste dump, domestic wastewater, phosphate fertilizer production, wood production, and fossil fuel combustion. Cu can accumulate in aquatic organisms and transfer through the aquatic food chain. Although Cu is an essential element in human physiological function, exposure to excessive levels of Cu can cause liver and kidney damage, the destruction of red blood cells, immunotoxicity, and developmental toxicity [67,68]

Hg occurs naturally in the Earth’s crust in several forms, and has been used in several industrial production processes [69]. It can be found in metal forms such as mercury salts or as organic mercury compounds such as methylmercury (MeHg). Once Hg is released to the environment, it will finally end up in soil, water, and sediment. In aquatic environments, Hg can be transformed to methylated by bacteria to MeHg, and accumulate in aquatic food chains through bioaccumulation and biomagnification processes. Human exposure to Hg can cause adverse effects such as nervous system damage, DNA damage, and negative reproductive effects such as birth defects [70].

Mn is naturally present in rocks or soils. It has been used for steel production, aluminum alloy, dried cell battery paints, and fertilizer. Mn is water soluble, where Mn^+2^ is the dominant form. Although Mn is an essential element for humans, high levels of exposure via inhalation can cause adverse effects to the central nervous system (CNS), while adverse effects of exposure via oral routes are limited [71].

Ni is a natural element in the Earth’s crust. It has been used in several applications such as alloys, electroplating, batteries, coins, machinery parts, nickel-chrome resistance wires, and catalysts. Ni is an essential nutrient for some mammals and humans; however, excessive exposure to Ni via inhalation and dermal contact might cause adverse effects such as lung inflammation, nasal cavity damage, and allergic dermatitis, while toxic effects via oral exposure are unlikely to occur [72].

Pb occurs naturally in the Earth’s crust. It has been used in batteries, metal products, ammunition, paint, and ceramic products. Pb particles disperse in the air and are removed by wet or dry deposition, while Pb compounds in wastewater can be released to the river and accumulate in water, sediment, and aquatic organisms. Adverse effects of Pb exposure via ingestion can produce gastrointestinal symptoms, colic, constipation, abdominal pain, anorexia, and vomiting, while chronic Pb exposure can cause adverse effects on blood pressure, kidney function, and immune system [73]. The major concern of Pb exposure is in relation to its effects on IQ, learning, memory, and the behavior of children who are exposed to low levels of Pb in their early life.

Zn is one of the most common elements in the Earth’s crust. It has been used in various industries such as coating steel for construction, coating metal products, metal plating, wood preservative, dry batteries, textile machines, printers, paints, and pesticides. Zn is an essential nutrient for the human body; however, exposure to high doses can affect leukocyte numbers and functions, and decrease the density of lipoprotein (HDL) levels [74].

**Figure 2:**
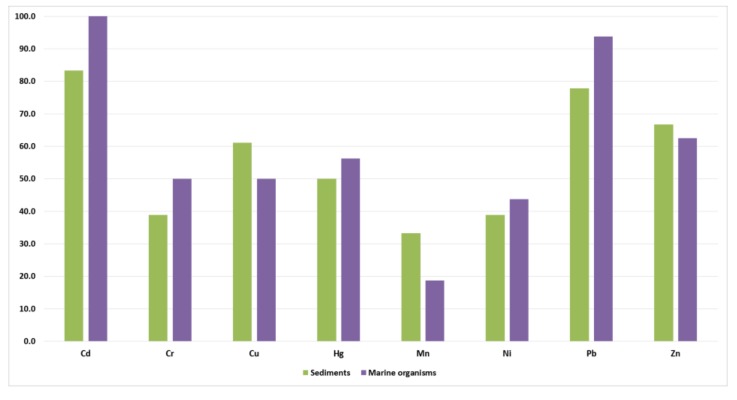
Percentage of reviewed documents that determined each trace element.

Overall, for the articles acquired in this review, Cd was found in most of the sediment samples, followed by Pb, Zn, Cu, Hg, Cr, Ni, and Mn, respectively. Meanwhile, Cd was also determined in most of marine organism samples, followed by Pb, Zn, Hg, Cr, Cu, Ni, and Mn, respectively (Figure 2).

## 5. Discussion and Conclusions

Excessive land-based pollution caused by population growth, urbanization, and industrialization along the coastline is the main driving force of marine environmental degradation in the Gulf of Thailand [75]. Most studies on trace elements in the Gulf ecosystems have focused on the Upper Gulf region, and especially the major river estuaries and the Eastern Coast, where industrial activities have increased. The most-studied marine organisms have been blood cockles, green mussels, and oysters; Hg was typically the metal of concern.

The highest concentrations of Cd, Cr, Hg, Pb, and Zn in marine sediment throughout the reviewed data were 3.37 mg/kg [5], 82.61 mg/kg [24], 14 mg/kg [25], 79.8 mg/kg, and 800 mg/kg [5], respectively. Meanwhile, in marine organisms, the highest concentration of Cd, Cr, Cu, Hg, Pb, and Zn were found in *A. granosa* (21.14 mg/kg), *A. granosa* (5.21 mg/kg) [51], *A. granosa* (46.25 mg/kg) [60], *Orcaella brevirostris* (64.39 mg/kg) [51], *A. granosa* (11.09 mg/kg) [60], and *Amusium pleuronectes* (576 mg/kg) [57], respectively. The sediment and marine organisms from the Upper Gulf exhibited higher levels of trace elements than those from the Lower Gulf. This might be because the semi-enclosed condition of the Upper Gulf limits water exchange, leading to a higher accumulation of environmental pollutants than the Lower Gulf [15].

Spatial and temporal variations in trace element contents were observed, which were most likely related to anthropogenic activities [76] such as agriculture, aquaculture, tourism, urbanization, industry, harbor operations, and oil spills [5,14,36,77,78]. Other factors affecting the levels of trace elements in sediment and marine organisms included the input and transport of metals by river runoff, proximity to the sources of contamination, sediment composition, salinity, and meteorological conditions [79].

Marine bivalves are considered good sentinel species for trace element contamination, as they are bottom feeders or filter feeders [80,81,82]. In most of the studies reviewed here, levels of trace elements did not exceed permissible limits; however, the periodic monitoring of marine environments should be performed for preventing adverse environmental and health effects.

## Figures and Tables

**Table 1:** Concentrations of trace elements in marine sediment of the Gulf of Thailand.

Study Period	Study Area	Concentration Reported (µg/g Dry Weight)	Ref.
1984	Upper Gulf of Thailand	Cd (0.002); Co. (0.0098); Pb (0.0065) ^a^	[20]
1998–2001	Chao Phraya Estuary	As (0.012); Cd (0.0002); Hg (0.0018) ^a^	[26]
1999	Upper Gulf of Thailand		[21]
	- Pre-monsoon	Al (4.38); Cd (0.42); Cr (85.0); Cu (19.7); Fe (2.13); Pb (16.2); Zn (61.1) ^a^	
	- Post-monsoon	Al (5.34); Cd (0.35); Cr (62.7); Cu (25.7); Fe (1.22); Mn (368); Pb (29.9); Zn (51.6) ^a^	
-	Northwestern Gulf of Thailand	Cr (27.21–1104.60); Cu (7.53–363.64); Mn (0.33–12.27); Ni (10.09–885.24); Zn (25.98–1454.78) ^b^	[27]
2003	Coastal area of the Gulf of Thailand	Cu (1500–4000) ^b^	[22]
2004	Eastern Coast of the Gulf of Thailand	Cd (<0.006–0.19); Cu (14.4–103); Hg (0.005–0.121); Mn (30–1,710); Ni (<0.64–79.9); Zn (7.48–131) ^b^	[5]
2006	Pattani Bay		[38]
	- Mangrove + Topsoil	As (2.18); Cd (0.63); Hg (0.0073); Pb (41.70); Zn (19.54) ^a^	
	+ Subsoil	As (2.03); Cd (0.42); Pb (7.74); Zn (19.02) ^a^	
	- Pattani river mouth + Topsoil	As (0.33); Cd (0.15); Hg (0.0066); Pb (557.15); Zn (40.89)^a^	
	+ Subsoil	As (4.75); Cd (0.21); Pb (11.04); Zn (11.42) ^a^	
	- Pattani river + Topsoil	As (2.25); Cd (0.09); Hg (0.0041); Pb (9.20); Zn (8.07) ^a^	
	+ Subsoil	As (2.23); Cd (0.22); Pb (7.63); Zn (12.27) ^a^	
	- Industrial zone + Topsoil	As (0.89); Cd (0.31); Hg (0.1570); Pb (69.49); Zn (11.93) ^a^	
	+ Subsoil	As (3.48); Cd (0.20); Pb (5.01); Zn (16.03) ^a^	
	- Dockyard A + Topsoil	As (4.76); Cd (0.42); Hg (0.0142); Pb (358.77); Zn (9.01) ^a^	
	+ Subsoil	As (3.18); Cd (0.81); Pb (19.36); Zn (9.01) ^a^	
	- Dockyard B + Topsoil	As (2.19); Cd (0.27); Hg (0.0056); Pb (50.15); Zn (29.94) ^a^	
	+ Subsoil	As (2.03); Cd (0.38); Pb (4.27); Zn (14.42) ^a^	
2007	Upper Gulf of Thailand	Cd (2.05); Cu (16.21); Pb (28.19); Zn (64.07) ^a^	[30]
2009	Bang Ya Praeg, Tha Chin Estuary, Samut Sakhon Province	Cd (0.035–0.070); Cu (7.90–21.91); Pb (11.91–25.74); Zn (55.99–75.05) ^a^	[35]
2011–2012	Western Gulf of Thailand	As (1.06–34.07); Cd (0.02–0.17); Cr (8.08–82.61); Cu (2.20–25.31); Hg (0.00–0.11); Pb (4.13–38.75); Zn (1.63–78.95) ^b^	[24]
2012	Inner Gulf of Thailand	As (0.09–0.27); Cd (<0.003–0.07); Cu (0.7–16.0); Hg (<0.0005–0.12); Pb (2.6–38.0); Zn (1.2–2.5) ^b^	[40]
2012	Coastal area of Chanthaburi Province	Cd (0.018 ± 0.005); Cr (8.644 ± 1.648); Cu (7.414 ± 1.952); Pb (1.818 ± 0.525); Zn (18.122 ± 3.367) ^c^	[23]
2012–2013	Laem Bhak Bia, Phetchburi Province	As (3.280); Cd (0.023); Cr (10.454); Hg (0.015); Ni (2.963); Pb (18.831) ^a^	[42]
2012–2013	Laem Bhak Bia, Phetchburi Province	As (3.280); Cd (0.023); Cr (10.454); Hg (0.015); Ni (2.963); Pb (18.831) ^a^	[42]
-	Don Hoi Lot sandbar, Samut Songkhram Province	Cd (0.85 ± 0.28); Fe (5806.53 ± 333.51); Mn (409.40 ± 26.23); Ni (8.05 ± 0.28); Pb (25.84 ± 0.68) ^c^	[33]
2014	Lam Chabang, Chonburi Province	Cd (0.018–3.37); Cu (2.69–126); Hg (0.04–0.325); Ni (2.16–26.8); Pb (14.9–79.8); Zn (28.2–800) ^b^	[36]
2015–2016	Bangtaboon Estuary, Phetchburi Province	Cd (0.02–0.33); Cr (34.00–65.81); Cu (10.15–43.10); Hg (0.05–0.09); Mn (2090.00–3865.00); Ni (14.84–26.62); Pb (0.59); Zn (13.69) ^b^	[39]
	Tha Chin Estuary, Samut Sakhon Province	Cd (0.01–0.2); Cr (34.22–70.09); Cu (17.15–37.88); Hg (0.03–0.08); Mn (1250.00–5585.00); Ni (13.84–38.44); Pb (1.14–24.45); Zn (53.45–131.40) ^b^	
	Muaeng Chonburi, Chonburi Province	Cd (0.05–0.23); Cr (29.38–56.97); Cu (14.31–22.46); Hg (0.06–0.09); Mn (1345.00–2235.00); Ni (14.74–38.20); Pb (0.17–31.58); Zn (65.10–101.78) ^b^	
2016	Don Hoi Lot sandbar, Samut Songkhram Province	Cd (0.86 ± 0.05); Fe (5891.07 ± 61.72); Mn (499.20 ± 16.18); Ni (8.205 ± 0.1); Pb (25.81 ± 0.18) ^c^	[34]
2016	Eastern coast of the Gulf	As (<62.7); Cr (1.3–126); Cu (<29); Pb (21.3–134.0); Zn (2–103) ^b^	[41]
	Inner Gulf	As (0.4–16.6); Cr (12.6–157); Cu 5.8–37); Pb (23.9–163.0); Zn (17.2–140) ^b^	

Remark: ^a^ = average; ^b^ = range; ^c^ = mean ± SD; Hg = Total Hg.

**Table 2:** Concentrations of trace elements in marine organisms of the Gulf of Thailand.

Study Period	Study Area	Organisms	Concentration Reported (µg/g Wet Weight)	Ref
1982	Ang Sila bay	Blood cockle (*A. granosa*)	Cd (1.88–3.71); Cr (0.14–0.28); Cu (1.36–2.44); Pb (1.64–2.77); Zn (79.15–119.81) ^b,^**	[44]
		Green mussel (*P. viridis*)	Cd (0.09–2.51); Cr (0.01–2.17); Cu (0.66–17.92); Pb (0.19–3.80); Zn (58.27–285.70) ^b,^**	
		Oyster (*C. commercialis*)	Cd (0.33–7.44); Cr (0.15–0.20); Cu (4.89–276.30); Pb (0.54–3.80); Zn (64.11–1920) ^b,^**	
1989–1990	Coast of Thailand	Green mussels (*Perna viridis*)	Cd (<0.006–0.19); Cu (14.4–103); Hg (0.005–0.121); Mn (30–1,710); Ni (<0.64–79.9); Zn (7.48–131) ^b,^**	[46]
1995	Upper Gulf of Thailand	Green mussels (*Perna viridis*)	Cd (1.48 ± 0.23); Cr (0.97 ± 0.11); Cu (10.48 ± 1.05); Pb (1.26 ± 0.30); Zn (95.36 ± 10.39); Mn (3.02 ± 0.28); Ni (1.74 ± 0.19) ^c,^**	[48]
	Lower Gulf of Thailand	Green mussels (*P. viridis*)	Cd (0.92 ± 0.12); Cr (0.59 ± 0.19); Cu (9.51 ± 0.73); Pb (0.90 ± 0.159); Zn (91.09 ± 11.91); Mn (2.68 ± 0.39); Ni (1.34 ± 0.21) ^c,^**	
1999	Phetchaburi Province	Edible sea animals	Cd (0.229); Cu (0.434); Pb 910.382); Zn (36.444)	[49]
1999	Bangpakong estuary, Chonburi Province	Blood cockle (*A. granosa*)	Cd (0.275–0.880); Hg (0.244–0.366); Pb (0.180–0.328); Zn (74.304–93.300) ^b,^**	[50]
	Bangboea, Samut Prakan Province	Blood cockle (*A. granosa*)	Cd (0.237–0.388); Hg (0.250–0.343); Pb (0.150–0.198); Zn (82.768–99.673) ^b,^**	
	Klongtamru, Chon Buri Province: Farm	Blood cockle (*A. granosa*)	Cd (0.272–0.746); Hg (0.228–0.559); Pb (0.162–0.213); Zn (47.244–81.493) ^b,^**	
	Klongtamru, Chon Buri Province; Naturally grown	Blood cockle (*A. granosa*)	Cd (0.275–0.526); Hg (0.231–0.380); Pb (0.125–0.286); Zn (79.309–100.76) ^b,^**	
2007–2008	Map Ta Phut Industrial Estate, Rayong Province	Fish	Cd (<0.005–0.091); Cu (<0.022–0.96); Hg (<0.004–0.185); Pb (<0.005–0.41); Zn (0.40–13.6) ^b,^**	[57]
		Shrimp	Cd (<0.005–0.090); Cu (3.91–11.9); Hg (0.005–0.087); Pb (<0.005–0.098); Zn (14.6–38.3) ^b,^**	
		Crayfish	Cd (0.012–1.76); Cu (3.96–478); Hg (<0.004–0.083); Pb (<0.005–0.21); Zn (8.15–382) ^b,^**	
		Mollusc	Cd (0.181–5.23); Cu (0.091–1.21); Hg (<0.004–0.024); Pb (0.022–0.533); Zn (4.43–576) ^b,^**	
2008	Ban Leam, Phechaburi Province	Blood cockle (*A. granosa*)	Cd (0.72–21.14); Cr (<0.47–5.21); Ni (0.56–9.43); Pb (0.17–3.91) ^b,^*	[51]
2009	Phetchaburi Province/Samut Sakhon Province/Samut Songkram	Blood cockle (*A. granosa*)	Cd (≤0.05–1.23); Hg (ND–0.04); Pb (<0.12–0.75) ^b,^**	[54]
2011	Coast of Songkla province	Marine organisms	Cd (0.001–3.017); Cu (0.194–50.309); Pb (0.011–2.814); Zn (0.765–32.806) ^b,^**	[58]
2012	Chanthaburi Province	Oysters (*S. cucullata*)	Cd (0.752 ± 0.193); Cr (0.201 ± 0.051); Cu (32.577 ± 5.860); Pb (0.011 ± 0.003); Zn (186.180 ± 10.688) ^c,^*	[23]
		Green mussels (*P. viridis*)	Cd (0.093 ± 0.023); Cr (0.161 ± 0.033); Cu (10.039 ± 4.224); Pb (0.010 ± 0.002); Zn (31.472 ± 4 4.084) ^c,^*	
2012	Trat Province	Hard shall calm (*M. casta*)	Cd (0.00201); Cr (0.00375); Hg (0.00003); Ni (0.00856); Pb (0.00080) ^a,^**	[53]
		Hard shall calm (*M. meretrix)*	Cd (0.00338); Cr (0.00162); Hg (0.00003); Ni (0.00294); Pb (0.00051) ^a,^**	
		Razor calm (*S. grandis*)	Cd (0.00108); Cr (0.00170); Hg (0.00003); Ni (0.00275); Pb (0.00102) ^a,^**	
		Razor calm (*S. regularis*)	Cd (0.00095); Cr (0.00328); Hg (0.00003); Ni (0.00737); Pb (0.00191) ^a,^**	
		Razor calm (*S. strictus*)	Cd (0.00059); Cr (0.00066); Hg (0.00003); Ni (0.00106); Pb (0.00032) ^a,^**	
		Mollusc *(Pseudodon* sp.)	Cd (0.00010); Cr (0.00247); Hg (0.00003); Ni (0.00245); Pb (0.00169) ^a,^**	
2012–2013	Phetchaburi Province	*Blood cockle (A. granosa)*	As (0.00917); Cd (0.00577); Cr (0.00060); Hg (0.00009); Ni (0.00175); Pb (0.00089) ^a,^**	[52]
		Hard shall calm (*K. hiantina)*	As (0.01458); Cd (0.00151); Cr (0.00092); Hg (0.00011); Ni (0.00268); Pb (0.00067) ^a,^**	
		Hard shall calm (*K. Marmorata)*	As (0.04037); Cd (0.00532); Cr (0.00749); Hg (0.00034); Ni (0.00759); Pb (0.00242) ^a,^**	
		Hard shall calm (*M. hiantina)*	As (0.00809); Cd (0.00112); Cr (0.00059); Hg (0.00007); Ni (0.00207); Pb (0.00036) ^a,^**	
		Hard shall calm (*M. Marmorata)*	As (0.02139); Cd (0.00273); Cr (0.00152); Hg (0.00018); Ni (0.00510); Pb (0.00091 ^a,^**	
		Hard shall calm (*P. attenuates*	As (0.01044); Cd (0.00049); Cr (0.00128); Hg (0.00011); Ni (0.00391); Pb (0.00144) ^a,^**	
2012–2013	Phetchaburi Province	Hard shall calm (*M. casta*	Cd (0.00049); Cr (0.00055); Hg (0.00013); Ni (0.00222); Pb (0.00050) ^a,^**	[42]
		Hard shall calm (*M. meretrix*)	Cr (0.00116); Hg (0.00019); Ni (0.00412); Pb (0.00226) ^a,^**	
2013–2014	Samut Songkhram Province	Green mussels (*P. viridis*)	Cd (0.10–0.12); Hg(ND); Pb (<0.50) ^b,^**	[56]
	Pethchaburi Province	Green mussels (*P. viridis*)	Pb (ND–0.73) ^b,^**	
	Samut Sakhon Province	Green mussels (*P. viridis*)	Cd (<0.10–0.11); Hg(ND–<0.008); Pb (ND–<0.05) ^b,^**	
	Samut Songkhram Province	Blood cockle (*A. granosa*)	Cd (0.15–1.15); Hg (0.01–0.07); Pb (ND–<0.50) ^b,^**	
	Pethchaburi Province	Blood cockle (*A. granosa*)	Cd (0.17–0.61); Hg (ND -0.01); Pb (<0.50–2.15) ^b,^**	
	Samut Sakhon Province	Blood cockle (*A. granosa*)	Cd (0.86–1.92); Hg (<0.008–0.01); Pb (ND–<0.50) ^b,^**	
2014	Pattani Bay, Pattani Province	Blood cockle (*A. granosa*)	Cd (0.02–7.58); Cu (0.13–46.25); Pb ((0.08–11.09); Zn (1.01–117.29) ^b,^*	[60]
2015–2016	Bangtaboon Estuary, Phetchburi Province	Blood cockle (*A. granosa*)	Cd (0.35–0.45); Cr (0.57–1.01); Cu (4.13–7.14); Hg (0.03–0.04); Mn (34.10–59.10); Ni (0.34–0.85); Pb (0.09–0.25); Zn (82.55–82.92) ^b,^*	[61]
	Tha Chin Estuary, Samut Ssakhon Province	Blood cockle (*A. granosa*)	Cd (1.56–4.24); Cr (0.40–0.88); Cu (2.60–4.62); Hg (0.02–0.05); Mn (29.10–176.40); Ni (0.53–0.46); Pb (0.05–0.46); Zn (39.75–84.81) ^b,^*	
	Muaeng Chonburi, Chonburi Province	Blood cockle (*A. granosa*)	Cd (3.16–4.52); Cr (0.19–0.46); Cu (2.90–4.88); Hg (0.03–0.04); Mn (13.00–23.00); Ni (0.46–1.29); Pb (0.19–0.36); Zn (34.12–70.03) ^b,^*	

Remark: ^a^ = average; ^b^ = range; ^c^ = mean ± SD; * dry-weight; ** wet weight; Hg = Total Hg.
